# A scoping review of the national strategy for brucellosis control in Egypt: logic framework, challenges, and prospects

**DOI:** 10.1186/s42522-025-00168-2

**Published:** 2025-09-10

**Authors:** Ekram W. Abd El-Wahab

**Affiliations:** Present Address: Department of Tropical Health, High Institute of Public Health, Alexandria University, 165 El Horreya Road, Alexandria, 21561 Egypt

**Keywords:** Brucellosis, Control, Egypt, Logic framework

## Abstract

**Background:**

Brucellosis remains a significant public health and economic challenge in Egypt despite long-standing control efforts. This paper outlines the national strategy for brucellosis control, detailing its legal framework, diagnostic protocols, surveillance mechanisms, vaccination programs, and biosecurity measures.

**Main body:**

Egypt employs a dual approach of test-and-slaughter and selective vaccination, supported by serological and pathological diagnostics. Surveillance combines passive reporting, risk-based monitoring, and active outbreak investigation. While progress has been achieved, particularly in establishing brucellosis-free dairy compartments, eradication remains elusive due to inconsistent compliance, limited animal registration, inadequate compensation, and cultural barriers. Challenges also include insufficient epidemiological data, especially in small ruminants, and weak coordination between veterinary and public health sectors. Global comparisons highlight the importance of ecosystem-based and One Health approaches.

**Conclusion:**

This review identifies critical gaps in surveillance, control coverage, and stakeholder engagement. It calls for integrated policy reforms, investment in diagnostic and monitoring infrastructure, enhanced public awareness, and regional cooperation to accelerate Egypt’s path toward brucellosis elimination.

## Graphical Abstract

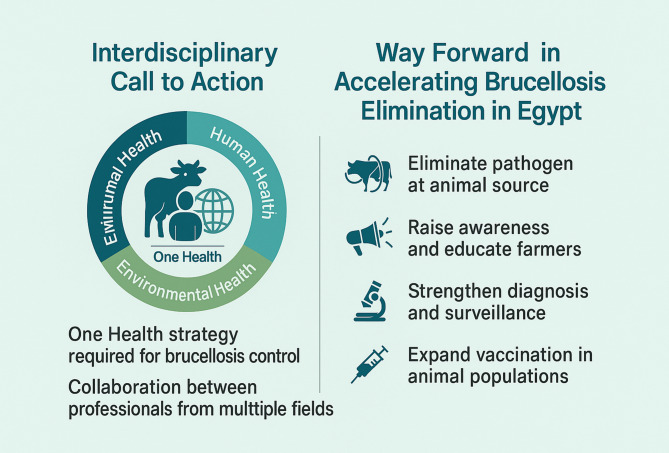

## Background

Brucellosis is the world’s leading neglected zoonosis, posing significant public health and economic burdens in many developing regions. While controlled in some countries, it remains endemic across the Mediterranean, Western Asia, Africa, and Latin America [1, 2]. Human infection primarily originates from livestock—cattle, buffaloes, sheep, and goats—harboring *Brucella* spp [3, 4]. , making animal-focused control strategies the most effective approach [5]. In animals, brucellosis causes abortion, stillbirth, mastitis, metritis, placental retention, orchitis, arthritis, and infertility in both sexes. It is caused by gram-negative coccobacilli of the genus *Brucella*, affecting various livestock species [6, 7]. Since the discovery of *Brucella melitensis* (*B. melitensis*) by David Bruce in 1887, multiple species have been identified, including *B. abortus* (cattle), *B. melitensis* (sheep and goats), *B. suis*, *B. ovis*, *B. canis*, *B. neotomae*, *B. pinnipediae*, and *B. cetaceae* [7, 8].

## Transmission and risk factors

Brucellosis, designated by the World Health Organization as a neglected zoonosis and a risk group III pathogen, poses significant public health threats due to its ease of aerosol transmission [9]. Airborne infection with *B. melitensis* has been documented, and *Brucella* spp. have been utilized as biological weapons in the past. The disease is commonly transmitted through consumption of contaminated milk and dairy products, direct skin contact during animal husbandry, and accidental exposure in laboratory or clinical settings [6]. Occupational exposure represents a major risk, particularly among farmers, slaughterhouse workers, meat processors, dairy handlers, butchers, veterinarians, and laboratory personnel, who may become infected through inhalation of aerosols, skin inoculation, or handling infected animals during parturition, delivery of placenta, or dealing with aborted animals.

Consumption of unpasteurized dairy products such as traditional village cheese or ice cream from street vendors is a frequent source of infection. In Egypt, social misconceptions regarding the nutritional value of pasteurized milk have significantly reduced its consumption, leading to a reliance on raw milk from small-scale producers, which now supplies up to 80% of the country’s milk—approximately 4 billion liters annually [10]. This is compounded by the widespread distribution of food products through informal markets that often lack proper health and safety oversight, particularly in low- and middle-income countries [11]. Eating raw or undercooked meat and engaging in hunting activities further elevate the risk of transmission.

Though rare, human-to-human transmission has been reported through blood transfusion, organ transplantation, sexual contact, artificial insemination, and breastfeeding. Congenital transmission can also occur via the placenta or breast milk. Exposure to live attenuated veterinary vaccines is another potential source of infection, particularly through accidental inoculation or inhalation [12–14]. Individuals who keep animals in their homes—especially small ruminants that are not vaccinated or have a history of abortion—face higher risks. Those involved in dairy product processing, who fail to use protective measures or neglect to report cases of brucellosis in humans or animals, are also at increased risk. Notably, the presence of another infected family member significantly raises the likelihood of household transmission. As such, screening the relatives of brucellosis patients may help identify additional undiagnosed cases [15].

## Magnitude of the disease

### Worldwide

Brucellosis remains a globally distributed zoonosis, but its true burden is likely underreported, particularly in regions with weak medical and veterinary infrastructure [16]. Although the disease has been documented worldwide, it is far more prevalent in countries lacking robust public health and animal disease control programs. High-risk regions include the Mediterranean Basin—such as Portugal, Spain, Southern France, Italy, Greece, Turkey, and North African countries—along with Mexico, Central and South America, Eastern Europe, much of Asia and Africa, the Caribbean, and the Middle East. Data from several Middle Eastern countries continue to highlight brucellosis as a significant public health issue [17].

In contrast, the World Organization for Animal Health (OIE) has declared that brucellosis has been eradicated or virtually eliminated from livestock in several countries, including those in Western and Northern Europe, Canada, Japan, Australia, and New Zealand. These achievements followed extensive and costly control programs [18]. Nevertheless, eradication remains a challenge even for developed countries such as the United States, primarily due to the ability of some *Brucella* species to infect multiple host species and persist in feral animal populations, particularly wild ruminants and swine [19].

The disease continues to affect high-risk occupational groups at notable rates. Studies have shown an average brucellosis prevalence of 11% among veterinarians, livestock handlers, and abattoir workers. Among hospital patients whose symptoms were clinically compatible with brucellosis, the prevalence was reported at 7% [20]. Moreover, in certain settings, brucellosis may account for as much as 30% of cases admitted with pyrexia of unknown origin [15].

### Egypt

Brucellosis was first reported in Egypt in 1939 and continues to pose a major threat to both animal health and human well-being, with significant economic and public health burdens [4]. Egypt’s livestock sector includes mixed populations of sheep, goats, cattle, and buffaloes. While *B. melitensis* is historically associated with small ruminants, infections in cattle and buffaloes have been increasingly reported. During the 1960s to 1980s, the prevalence in cattle reached 38%, particularly following the importation of foreign Friesian breeds into large-scale governmental farms. Notably high infection rates were observed at Tambesha Farm in Menofia and Dulla El Baraka in El Sharkia Governorate, the latter recording 71% positive and 14% suspicious cases, which hindered implementation of a test-and-slaughter policy [4].

This rise in animal infection was mirrored in the human population, with reported brucellosis cases escalating from 24 in 1988 to 1,429 by 1998, raising concerns about a potential epidemic. Animal-level seroprevalence has been reported at 5.4% in cattle, 4.1% in buffaloes, 5.4% in sheep, and 3.5% in goats [21], while other studies noted 7.02% prevalence in camels and 9.72% in swine. In humans, seroprevalence estimates have reached 11.0% (95% CI: 3.06–18.4%) at the individual level and 15.5% (95% CI: 6.61–24.7%) at the household level [22]. More recent findings have confirmed endemicity, with seroprevalence rates of 12.2% in sheep, 11.3% in goats, and 11% in cattle [22–25].

Despite the implementation of a national control program, brucellosis remains present in all Egyptian governorates, with up to 15% of livestock estimated to be seropositive in certain regions [26, 27]. Reliable national prevalence data are limited, with most estimates derived from small-scale seroprevalence studies. For example, a study in two villages in Gharbia governorate found no positive animals in one village, while the other reported a 16% seroprevalence [28]. The absence of comprehensive animal statistics and the widespread practice of household livestock keeping make it difficult to calculate true disease incidence [29].

Compounding this issue is the inconsistent use of diagnostic tests, with some studies relying on assays not recommended by WHO/OIE for the species tested. This results in potential overestimation due to low specificity, cross-reactivity, or inability to differentiate between vaccinated and infected animals [17]. Although brucellosis is a notifiable disease in Egypt, it remains largely underrecognized and underreported. Awareness among clinicians is low, and most public health laboratories do not routinely test for brucellosis. Consequently, many human cases go undiagnosed or are misclassified as pyrexia of unknown origin [4].

Reported human incidence rose from 0.5 per 100,000 population in 1994 to 1.9 per 100,000 in 1998 [4]. However, localized studies reveal much higher figures. In Fayoum Governorate, for example, the estimated annual incidence reached 64 cases per 100,000 in 2002 and 70 cases per 100,000 in 2003 [30]. These trends raise questions about the adequacy or implementation of Egypt’s official control program, despite its alignment with FAO/WHO/OIE guidelines.

Brucellosis among high-risk occupational groups also remains a concern. In one study, 31.3% of slaughterhouse workers tested positive using the standard agglutination test (SAT), confirmed by ELISA [31]. A population-based surveillance of acute febrile illness (AFI) in Fayoum during the summers of 2002 and 2003, involving all hospitals and a representative sample of community healthcare providers, identified 321 brucellosis cases among 4,490 enrolled patients—an overall rate of 7% [30].

Regarding the farm-versus household-level brucellosis status in animals, the household or smallholder farm is typically the primary epidemiological unit for brucellosis surveillance and control, as most livestock are raised in mixed-species herds by rural families [26, 32]. This makes the farm or household functionally equivalent to the herd level, which is critical for understanding disease dynamics. Although national surveillance once reported relatively low prevalence—around 0.9% in cattle, 0.3% in buffaloes, and up to 8.2% in small ruminant [23]—more recent targeted studies have identified seropositivity in 17–27% of herds, particularly in high-risk regions [22, 26]. Despite the existence of an official control program involving annual serological testing, culling of confirmed positives, and vaccination with S19 or Rev.1 [9], inconsistencies in implementation—such as incomplete herd testing and non-random sampling—have led to unreliable classification of brucellosis-free herds [4]. Additionally, farms may be prematurely declared free of infection after limited testing, allowing undetected cases to persist. This is particularly concerning given the close contact between humans and livestock in rural areas, which facilitates zoonotic transmission. Although human seroprevalence remains relatively low (2–3%), clustering of cases in households with infected animals has been documented [15]. For effective national control, it is essential to clearly define brucellosis status at the herd level. We recommend that herds be initially classified as infected and only reclassified as disease-free after multiple consecutive rounds of negative testing. This would improve disease detection, strengthen certification processes, and better align local efforts with Egypt’s broader brucellosis elimination strategy.

## Impact of brucellosis at the human–animal interface

Brucellosis causes significant economic and public health burdens in endemic areas. In animals, it leads to abortions, reduced milk production, decreased weight gain, infertility, and premature culling, all of which diminish herd productivity, food security, and farmers’ livelihoods. Additional costs include veterinary services, vaccination, diagnostics, indemnification, and infrastructure for disease surveillance and control [33]. Trade restrictions due to endemic *Brucella* species, such as *B. melitensis*, *B. abortus*, and *B. suis*, further exacerbate economic losses [33].

Human brucellosis, marked by acute and chronic illness, results in significant work absenteeism and reduced income, thereby affecting household socioeconomic status. It also imposes direct healthcare costs (diagnosis, treatment, hospitalization) and indirect costs related to morbidity and mortality, such as disability-adjusted life years (DALYs) [33, 34]. Non-healthcare expenses include transportation and lost productivity, while intangible costs involve diminished quality of life and emotional distress, particularly in farming communities.

## Control of brucellosis

Effective public policy implementation is crucial to reduce the socioeconomic burden of brucellosis in both human and animal populations. Evidence indicates that strategic vaccination of ruminant livestock, supported by continuous surveillance and public health education, constitutes the most effective control strategy [26, 35]. Currently, there are no human vaccines available that are both safe and effective against Brucella infection [36].

## National policy for controlling brucellosis in Egypt

**Fig. 1:**
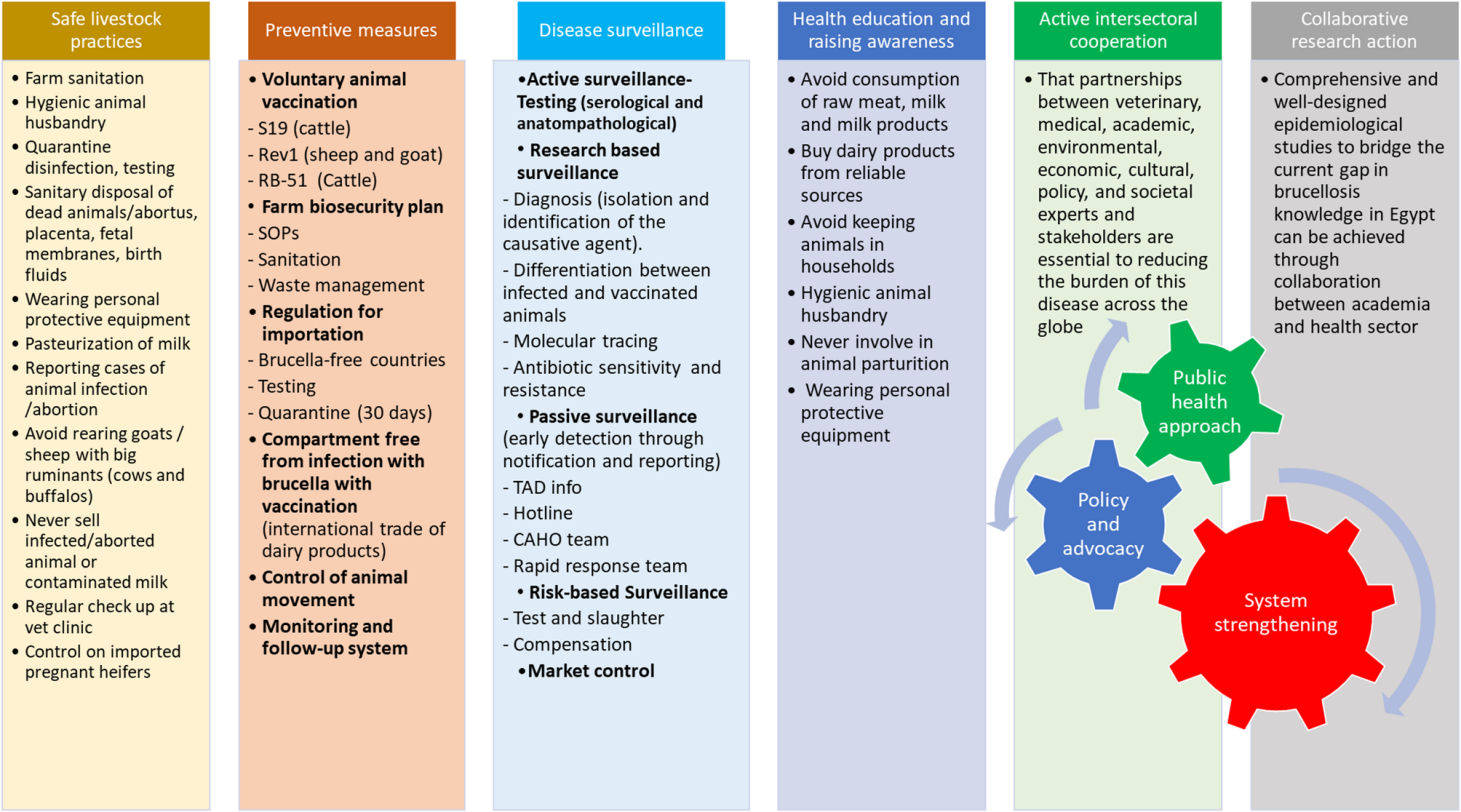
Key components of the National Brucellosis Control Program in Egypt (FAO/WHO/OIE). Overall aim is to control the animal brucellosis and to mitigate the risk of transmission of brucellosis to human

The national policy for brucellosis control in Egypt is enforced through several ministerial decrees, beginning with Decree No. 1067 of 1988, which sets standards for controlling brucellosis and bovine tuberculosis. This legislation outlines procedures including notification, testing, quarantine, slaughter, compensation, and vaccination schedules. Control measures involve a test-and-slaughter approach, sanitary disposal of positive animals, and restrictions on consumption of products from suspected cases (Fig. 1). The policy emphasizes comprehensive testing, slaughter, compensation mechanisms, and protective interventions aimed at reducing transmission and ensuring food safety [37].

### Tests for surveillance of brucellosis

Egypt’s compulsory national program for brucellosis control integrates two key strategies. The first involves serological surveillance conducted in the field by official veterinarians, who utilize the Buffered Acidified Plate Antigen (BAPA) and Rose Bengal tests. Seropositive samples are subsequently confirmed by complement fixation testing at the OIE Reference Laboratory within the Brucellosis Research Department, Animal Health Research Institute (AHRI). Testing targets cattle and buffaloes older than six months, which are screened biannually, as well as heifers that have aborted or exhibit clinical signs suggestive of Brucella infection.

The second strategy includes anatomopathological examinations and laboratory diagnostics. Samples such as placenta, fetal fluids, and blood serum—collected 14 to 21 days post-abortion—are submitted to the Brucellosis Research Department at AHRI. Confirmation includes culturing and isolation of Brucella from tissues such as the head, mammary glands, genital lymph nodes, and spleen obtained from slaughtered animals.

### Surveillance of brucellosis

The Brucellosis Research Department (BRD), in collaboration with veterinary authorities, conducts all laboratory activities related to brucellosis surveillance. These include the application and validation of serological tests, isolation and identification of Brucella at the genus, species, and biovar levels, differentiation of infected from vaccinated animals, molecular tracing of strains to determine source and origin, and assessment of antibiotic susceptibility and resistance profiles.

### Passive surveillance (reporting system)

Passive surveillance remains the primary method for early disease detection and outbreak warning. All relevant stakeholders must report suspected cases to the nearest veterinary clinic, which relays information to local and central authorities under the General Organization for Veterinary Services (GOVS). Supporting mechanisms include the Transboundary Animal Disease Information System (TAD info), a hotline operated by GOVS, and notification alerts via email, fax, and monthly reports on zoonotic diseases from all governorates. Community-Based Animal Health and Outreach (CAHO) teams, consisting of trained veterinarians, conduct participatory disease surveillance in diverse settings, such as routine monitoring in high-density animal populations, enhanced surveillance in high-risk areas, and investigations triggered by reports from animal keepers.

Upon notification, rapid response teams are deployed to conduct epidemiological investigations, enforce control measures, and collect samples for laboratory confirmation. No suspected cases have been reported in brucellosis-free compartments during the past year. Current suspicion criteria include ownership or treatment of animals by confirmed human brucellosis cases, abortions with no mechanical cause, and nonspecific signs such as low productivity and anestrus. Positive field test results prompt confirmatory testing at AHRI. Confirmed positive cases lead to emergency slaughter under hygienic conditions and active surveillance to control further transmission.

### Risk-based surveillance

Risk-based surveillance aims to control brucellosis and prevent its transmission to humans by targeting high-risk populations. Approximately 10% of the high-risk animal population, equating to about 350,000 heads annually, is tested using serological methods. Surveillance focuses on farm and backyard sectors, particularly in geographical areas with high brucellosis prevalence or dense animal populations, including mixed backyard systems. Targeted animals include cattle, water buffaloes, sheep, and goats over six months old, with particular attention to females and breeding males.

### Measures to prevent and control brucellosis in Egypt

Vaccination is an optional but recommended measure guided by national policy. Female cattle and buffaloes aged four to seven months are vaccinated with *B. abortus* strain 19, while female sheep and goats of the same age group receive *B. melitensis* strain Rev.1. Some farms utilize the rough *B. abortus* strain RB-51 under official veterinary supervision, as it produces antibodies that do not interfere with conventional diagnostic tests. Vaccinated animals are retested one year post-vaccination [25, 38].

Biosecurity measures are promoted and supervised by GOVS in dairy compartments, assisting producers in implementing protocols that prevent disease outbreaks, monitor animal health, and respond effectively to health challenges. These biosecurity plans include detailed identification of farm data, appointment of a biosecurity coordinator, annual training and evaluation of farm personnel, implementation of security procedures, disinfection protocols, visitor regulation, and protocols related to access control, feed and water safety, waste management, pest control, carcass disposal, and product safety. Official veterinarians are responsible for evaluating the implementation of these plans.

Importation of bovine animals and related products is tightly regulated to ensure brucellosis-free status. Imported animals must be certified free of brucellosis, undergo negative testing prior to shipment, and be isolated for 30 days pre-shipment. Upon arrival, they undergo a 33-day official quarantine, with further serological testing performed by the official veterinary laboratory.

### Implementation of the “compartment free from infection with Brucella with vaccination” program in dairy cattle farms

This program supports Egypt’s dairy exports by certifying farms as free from brucellosis infection. Eligibility requires no reported cases of brucellosis within the past year. Certification involves two negative tests of all sexually mature animals, with the first test conducted at least three months after slaughter of any positive animal and the second six months later. Animals presenting clinical signs consistent with Brucella infection must be tested and confirmed negative. Indeed, conducting only two tests before declaring a farm ‘Brucella-free’ may be insufficient to ensure the complete elimination of the infection. To enhance the reliability of the certification process, we recommend that all farms enrolled in the program initially be classified as infected herds. This conservative approach would help ensure more rigorous monitoring, reduce the risk of undetected cases, and support a more robust foundation for long-term disease control.

Participating farms must maintain comprehensive administrative and technical records, comply fully with government regulations, and collaborate transparently with veterinary authorities for sampling and reporting. Notification to authorities prior to any animal movement is mandatory.

Surveillance within the program adheres to national standards, involving biannual testing to ensure continued freedom from infection. Infrastructure for biosecurity is thoroughly evaluated during farm inspections to determine program eligibility. Veterinary officials conduct both scheduled and unannounced visits to monitor compliance. Movement controls restrict animal trade to certified brucellosis-free farms, with all movements requiring valid certification and negative test results confirmed twice yearly under official supervision.

Brucellosis remains a notifiable disease nationwide. Since 1981, Egypt’s brucellosis control program has mandated permanent identification of all vaccinated animals. Over the last three years, surveillance of 42 dairy cattle herds across 14 compartments has detected no evidence of infection. Animals showing clinical signs consistent with brucellosis have tested negative, and no infections have been found in neighboring herds or flocks. Where cases have occurred, preventive and control measures have effectively contained outbreaks. The OIE Delegate for Egypt has declared these 14 dairy cattle compartments compliant with OIE standards for freedom from Brucella infection with vaccination.

## Gaps in different brucellosis control strategies in different countries

A recent meta-analysis reviewed brucellosis control and eradication programs across various countries, revealing that only 3 out of 23 achieved freedom from animal brucellosis after several decades of implementation. Vaccination and test-and-slaughter, alone or combined, effectively reduce prevalence, but supplementary measures are essential and must be properly executed for success. Despite the high resource demand, well-designed programs prove economically viable, though sustainability is critical due to risks of disease recurrence [39].

Another meta-analysis assessing brucellosis awareness across 22 countries, mainly in Asia and Africa, found insufficient knowledge about the disease. Overall awareness was 55.5%, with awareness of its zoonotic nature, transmission, and symptoms in humans and animals ranging between 28.4% and 41.6%. Friends and neighbors were the primary information sources. Given that occupationally exposed populations’ perceptions affect disease control strategies and adoption of preventive measures, enhancing awareness and detailed knowledge through media and health workers is urgently needed to improve control efforts and protect human health [40].

A stochastic mathematical model was developed to evaluate the impact of the current test-and-slaughter policy on brucellosis prevalence in Egypt’s small-ruminant population. The model simulated different regimes of testing and culling seropositive sheep and goats in a village. Results indicated that testing 5–15% of animals every 1, 3, or 5 years and removing positives would not significantly reduce disease prevalence. Under current implementation levels, brucellosis would persist endemically at rates above 8.5%. Unless prevalence is low (≤ 2%), vaccination alone or combined with test-and-slaughter should be considered.

Increasing the proportion of animals tested every 3 or 5 years led to only gradual prevalence decreases. Annual testing showed a steady decline, with prevalence dropping below 1% after four years if 80% of animals were tested annually. However, even testing 100% did not eliminate the disease completely, likely due to imperfect sensitivity and specificity of serological tests. These findings emphasize the need for sufficiently frequent testing and a high proportion of animals tested to effectively reduce brucellosis prevalence in small ruminants [26].

## Strengthening the national brucellosis control policy

Implementing the targeted measures grounded in the policy’s existing framework and addressing critical operational gaps (Table 1), can significantly enhance Egypt’s capacity to reduce transmission, certify disease-free herds, and achieve sustainable brucellosis elimination.

**Table 1:** Key gaps in the National policy for controlling brucellosis and proposed action

Gap	Proposed Action
Low testing and quarantine compliance	Deploy regionally representative surveillance, aim for ≥ 10% annual testing coverage, ensure consistent follow-up testing and enforcement of quarantine until documented herd clearance.
Diagnostic limitations	Upgrade to molecular diagnostics (e.g. AMOSPCR, culture + strain typing) in provincial labs, integrate periodic bacteriological confirmation alongside serology to detect carriers.
Lack of animal ID/movement control	Implement a national livestock identification and registration system; enforce strict controls at animal markets and transport checkpoints; trace sources of infection.
Weak vaccination uptake & economic disincentives	Roll out biennial mass vaccination of all mature animals using S19 and Rev.1 (both male & female), before breeding/lactation seasons; introduce fair compensation schemes and market access benefits for vaccinated herds to incentivize participation.
Insufficient awareness & One Health integration	Launch tailored awareness campaigns targeting rural households, veterinarians, farmers, abattoir workers and public health personnel; strengthen One Health coordination between ministries; enforce biosafety standards in abattoirs and laboratories.

### Ecosystem management

Brucellosis will only be successfully controlled when solutions are conceived and implemented at ecosystem scales appropriate to wildlife, rather than being limited to local scales or adapted from agricultural management plans. If interactions between humans, wildlife, and livestock can be effectively managed, the resulting strategies could serve as a model for sustainable policy development. Ecosystem management is inherently multifaceted; policies must be tailored to fit specific ecosystems, species, diseases, and stakeholders. Nonetheless, all approaches share common principles. As noted, ecosystem management integrates scientific knowledge of ecological relationships within a sociopolitical and value-based framework, aiming to protect native ecosystem integrity over the long term. Management must prioritize intergenerational sustainability rather than short-term deliverables [41].

### Monitoring and evaluation of the brucellosis control program in Egypt

Despite longstanding efforts, brucellosis remains endemic in Egypt, with higher infection rates in small ruminants compared to cattle and camels. The existing control interventions, although able to maintain relatively low infection rates, have not succeeded in eradicating the disease. Several critical factors must be addressed in designing future intervention programs.

A major shortcoming is the absence of current, detailed epidemiological studies, especially among small ruminants, indicating the need to reassess disease prevalence nationwide. Such data would inform policymakers in selecting alternative strategies. Surveillance programs must be enhanced and standardized to generate accurate seroprevalence estimates. Furthermore, permanent and efficient animal registration systems are essential to monitor and control livestock movement.

Funding must support sustained, mid- to long-term monitoring efforts, as many control programs begin with promising activities but are prematurely terminated without proper evaluation or transition planning. Depending on the prevailing prevalence, strategies such as test-and-slaughter, mass vaccination followed by selective slaughter, or depopulation should be considered.

Veterinary supervision during culling is necessary to ensure safe carcass disposal and prevent environmental contamination. Human infection risk is compounded by the widespread consumption of raw milk. Public education is vital to highlight the importance of pasteurization and the ineffectiveness of traditional souring methods in eliminating Brucella, which survives in milk fat.

Most existing data derive from suspected cases, inherently yielding high infection rates and failing to reveal the broader epidemiological picture. An active, effective surveillance system is thus essential to determine true incidence rates in both humans and animals and to identify related risk factors.

Although Egypt’s OIE Delegate declared 14 dairy cattle compartments comprising 42 herds as compliant with brucellosis-free status through vaccination, the overall eradication program remains ineffective. Numerous challenges have contributed to inconsistent progress.

There is a notable lack of reliable disease prevalence estimates for both humans and livestock, hampering the development of integrated, multi-sectoral policies. Additionally, brucellosis seroprevalence data in sheep are insufficient, necessitating well-designed observational studies to assess the true burden of disease. Weak communication among public health authorities, veterinarians, and stakeholders further impairs coordinated efforts. Compounding these issues is the poor compliance of livestock owners during testing campaigns.

Surveillance and reporting systems are underfunded, and the movement of small ruminants remains largely unregulated, with incomplete registration. Smallholders, who own over 70% of Egypt’s livestock, often maintain highly migratory flocks, complicating control measures. Modeling studies estimate that only 4–5% of animal stocks are covered by control programs, with testing disproportionately concentrated in certain districts such as Kafr El Sheikh, where government laboratories are based.

Low compensation rates discourage farmers from slaughtering infected animals, with only 0.2% of seropositive animals being culled. Diagnostic practices are often inadequate, and mixed-species husbandry, common in Egypt, facilitates cross-species transmission. Brucellosis is also propagated through large animal markets, where animals of unknown health status from various regions mix freely.

Cultural attitudes, such as emotional attachment to livestock and distrust of government interventions, reduce owner willingness to report disease or allow slaughter of infected animals. A robust veterinary infrastructure, including animal identification systems, must be established. Additionally, a nationwide survey to genotype circulating Brucella strains is urgently needed to clarify the epidemiological situation. Cultivation and biotyping capabilities should be expanded to all governorates to monitor program effectiveness and trace outbreaks. Future seroprevalence studies must adhere to rigorous scientific standards.

The open sale of infected animals remains a pressing concern, with such practices increasing the risk of herd-wide outbreaks. Veterinary oversight of markets and satisfactory compensation for animal owners are essential to prevent the sale of infected stock. Culling, rather than indiscriminate slaughter, should be mandated to ensure safe carcass disposal and reduce environmental contamination.

Control measures must be made mandatory, with the implementation of reasonable compensation frameworks to encourage compliance. Egypt already possesses the basic infrastructure for such programs, including trained public veterinarians and state laboratories capable of serological testing. Information technology systems and animal identification solutions already in use elsewhere can be adapted to Egypt’s specific needs [15, 20–22, 26, 27, 35, 37, 42].

A relevant economic model from Mongolia demonstrated that an investment of USD $8.3 million in livestock vaccination could result in a return of USD $26.6 million. This reflects both increased livestock productivity and reduced human healthcare costs, income losses, and coping expenditures. Such results underscore the value of holistic, multidisciplinary approaches that disrupt animal-to-human transmission and may serve as templates for managing other zoonotic diseases [43].

### Interdisciplinary call to action

The control of brucellosis necessitates a ‘One Health’ approach, which recognizes the intrinsic interconnectedness of human, animal, and environmental health. This paradigm emphasizes that improvements in any one domain are contingent upon progress across all three. Consequently, effective mitigation of brucellosis requires robust interdisciplinary collaboration among experts in veterinary science, human medicine, environmental health, economics, policy, and social sciences. These partnerships are essential not only to reduce the disease burden globally but also to realize the associated economic benefits.

**Fig. 2:**
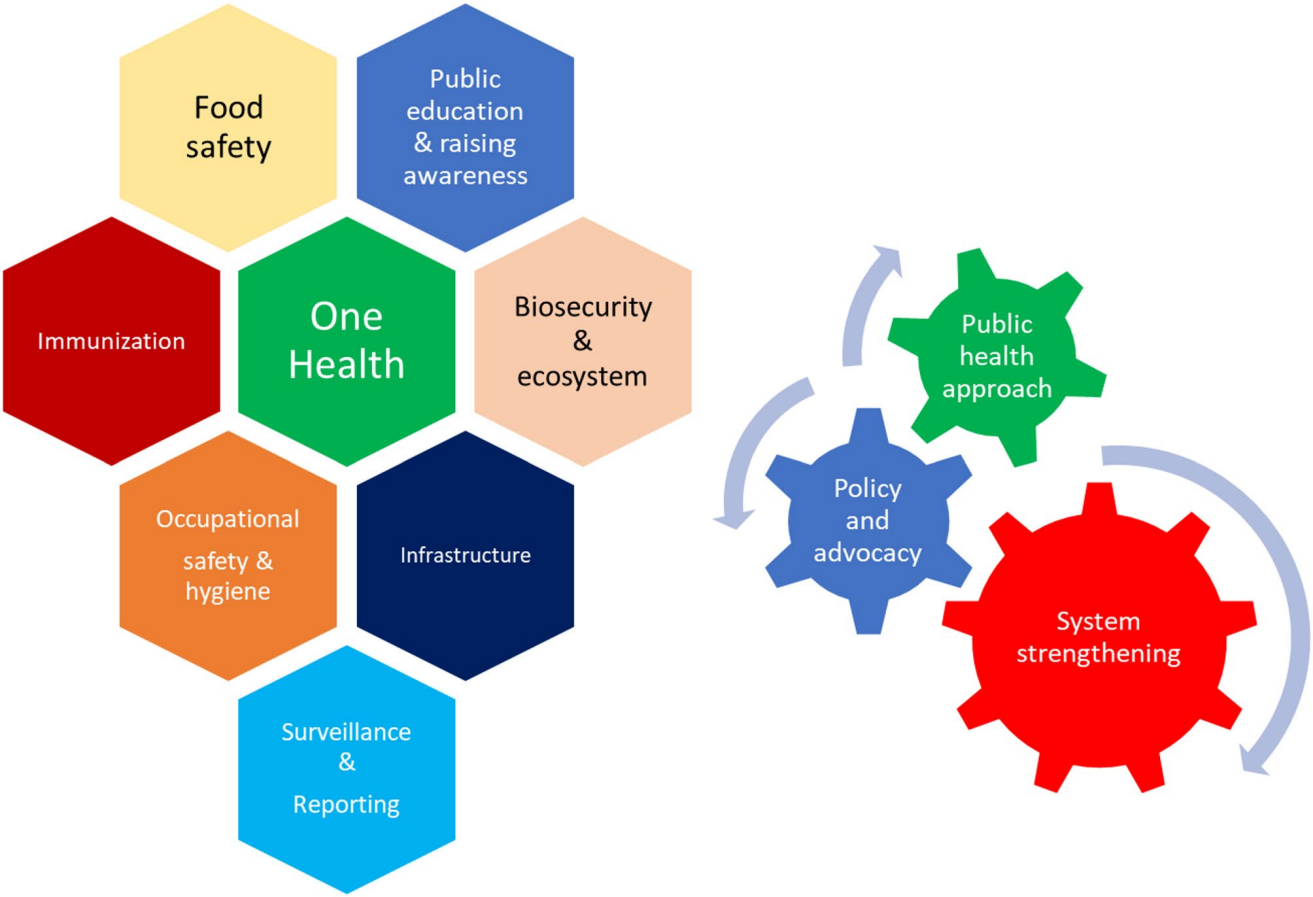
Logical Framework for a Brucellosis Control Program: Collaborative efforts from professionals across multiple disciplines and sectors

A collaborative, multisectoral strategy is required to prevent human brucellosis, including implementation of livestock vaccine campaigns, widespread community outreach, and public education to raise awareness about disease transmission and prevention. Food safety measures and occupational hygiene protocols, particularly in high-risk environments, must be emphasized. Laboratory biosafety standards should be improved, and a comprehensive infrastructure must be developed to support disease surveillance and reporting systems across both human and animal health sectors (Fig. 2). Furthermore, targeted control campaigns must be extended to wildlife and domestic animal populations alike, creating an integrated, long-term disease management strategy [33].

## Way forward in accelerating brucellosis elimination in Egypt

The first step in combating zoonoses such as brucellosis is to eliminate the pathogen at its animal source. Farmer awareness will play a crucial role in achieving this goal, and thus extensive education and outreach campaigns are essential. Strengthening the diagnostic infrastructure, including the establishment of a national OIE referral center for brucellosis in Egypt, will enhance early detection and timely intervention. Surveillance of human brucellosis, particularly at the human–animal interface, must be proactively implemented to monitor cross-species transmission dynamics.

Enhanced collaboration between human and veterinary research institutions is necessary to develop joint control strategies. Knowledge and capacity building among all stakeholders—including medical professionals, veterinarians, farmers, and the general public—should be prioritized. Encouraging safe animal handling practices, improving surveillance and reporting systems, and expanding vaccination coverage in small ruminants and other susceptible species are critical interventions.

All cases of animal abortions must be systematically reported and investigated to identify potential outbreaks. Diagnostic and laboratory testing capabilities must be expanded and strengthened, with national-level accreditation systems for laboratories conducting brucellosis research and diagnosis. This includes standardization and quality control of diagnostic kits, reagents, and vaccines.

A comprehensive, long-term approach is required, incorporating sustained investment, strategic program development, and regional collaboration with neighboring countries. Sound veterinary legislation and animal health policies must be implemented to support these efforts. Public awareness and health education, especially in rural communities, should be disseminated through diverse media channels and reinforced through the education of all personnel involved in disease control.

Prompt diagnosis, appropriate treatment, and proper case management in human patients are essential to avoid chronic complications and to support disease surveillance efforts. Regular dissemination of surveillance findings to relevant authorities and stakeholders will help refine ongoing control measures. Publicity and awareness campaigns must be launched at all administrative levels—from national to village level—and include training programs for state officials tasked with program implementation.

Animal identification, through ear-tagging and registration, should be mandated to facilitate monitoring. Maintenance of vaccination records through animal health cards will enhance traceability and program accountability. Regular serosurveillance and seromonitoring must be conducted across animal populations, and data must be generated and evaluated to measure the impact of interventions. Continuous monitoring and program assessment will be critical in guiding Egypt toward the successful elimination of brucellosis.

## Data Availability

No datasets were generated or analysed during the current study.

## Abbreviations

AHRI: Animal Health Research Institute
BRD: Brucellosis Research Department
CAHO: Community-Based Animal Health and Outreach
DALYs: Disability-Adjusted Life Years
ELISA: Enzyme-Linked Immunosorbent Assay
FAO: Food and Agriculture Organization
GOVS: General Organization for Veterinary Services
OIE: World Organization for Animal Health
SAT: Standard Agglutination Test
TAD info: Transboundary Animal Disease Information System
WHO: World Health Organization
